# Early-term outcomes of the pulmonary embolism response team

**DOI:** 10.12669/pjms.38.8.6541

**Published:** 2022

**Authors:** Yucel Ozen, Murat Ugur, Ismail Cihan Ozbek, Emre Yalcinkaya

**Affiliations:** 1 Yucel Ozen, MD. Department of Cardiovascular Surgery, University of Health Sciences, Sancaktepe Sehit Professor Doctor Ilhan Varank Education and Research Hospital, Istanbul, Turkey; 2 Murat Ugur, MD. Department of Cardiovascular Surgery, University of Health Sciences, Sancaktepe Sehit Professor Doctor Ilhan Varank Education and Research Hospital, Istanbul, Turkey; 3 Ismail Cihan Ozbek, MD. Department of Cardiovascular Surgery, University of Health Sciences, Sancaktepe Sehit Professor Doctor Ilhan Varank Education and Research Hospital, Istanbul, Turkey; 4 Emre Yalcinkaya, Department of Cardiology, University of Health Sciences, Sancaktepe Sehit Professor Doctor Ilhan Varank Education and Research Hospital, Istanbul, Turkey

**Keywords:** Catheter-Directed Thrombolysis, Pulmonary Embolism, Pulmonary Embolism Response Team, Systemic Thrombolysis

## Abstract

**Objective:**

Treatment of pulmonary embolism varies according to the different clinical presentations. Pulmonary embolism response teams (PERT) might improve outcomes of pulmonary embolism with faster evaluation and increased usage of advanced treatment methods. In this study, the effects of PERT for the treatment of pulmonary embolism were investigated.

**Methods:**

In this retrospectively analyzed study, patients diagnosed with PE in our hospital between March 1st, 2019 and February 28^th^, 2022 were included. Patients’ medical records were evaluated according to the treatment procedures and early outcomes.

**Results:**

Ninety-eight patients with pulmonary embolism were evaluated by the PERT during the study period. The mean age was 62.8+16.4 years and 59% were male. All patients with intermediate-low risk were treated medically. About 59.2% of the patients were hospitalized. The rate of catheter-directed thrombolysis was 37.8% (n=37). Systemic thrombolytic therapy was performed on two patients. One patient with a metastatic brain tumor was treated with low-molecular-weight heparin. Catheter-directed procedures were performed in 37 patients. The time from diagnosis to reperfusion was 243 minutes. There was one pericardial effusion and one mortality. In the 30-day follow- up there was no re-hospitalization and mortality.

**Conclusion:**

PERT might help early triage and treatment of patients with pulmonary embolism. Experienced specialists in this team might contribute to clinical recovery by performing advanced treatment methods and decreasing the risk of chronic thromboembolic pulmonary hypertension in the long term.

## INTRODUCTION

Pulmonary embolism is a life-threatening clinical condition with 32% mortality risk in case of hemodynamic instability.^1^ Most of the deaths occur due to massive pulmonary embolism (PE) and within one hour after the onset of clinical presentation.^1^ Pulmonary embolism, especially in the main pulmonary arteries, might be life-threatening and necessitates more aggressive treatment. Therefore, suspecting PE in the emergency service, detailed and advanced examination in suspected cases, and earlier treatment are the most important factors to improve outcomes of patients with PE.^2^

In the treatment of PE, exact treatment modalities and consensus have not been developed due to the differences in the clinical presentation of the patients regarding the involved arterial segment and the availability of different treatment methods. Therefore, Pulmonary Embolism Response Team (PERT) model in order to apply personalized treatment with a multidisciplinary approach has emerged.^3^ The first PERT was established in 2012 at Massachusetts General Hospital.^4^ PERT provides an environment where patients might be evaluated 24 hours a day, seven days a week. In addition to early diagnosis, PERT coordinates and accelerates risk assessment, management decisions, and treatment implementation.^5^,^6^

The aims of the treatment of PE are relieving obstruction by early reperfusion, regressing right ventricular dysfunction, and preventing progression to circulatory collapse.^4^ Pulmonary embolism might be treated conservatively by oral anticoagulants and by systemic thrombolysis (ST), or with advanced treatment options such as catheter-directed thrombolysis (CDT). Systemic thrombolysis and CDT prevent the progression of symptoms by re-canalization of the obstruction in the early period. Therefore, in the 2019 ESC treatment guideline, ST was advised as Class-1 indication and CDT are proposed as Class-2a.^7^ Also, it was stated that PERT, which facilitates the treatment plan and implementation, should be encouraged. Specialists forming the PERT might differ depending on the resources and experiences in each hospital.^5^ In this study, we evaluated the outcomes of the establishment of PERT in a new-built training and research hospital.

## METHODS

**Fig.1:**
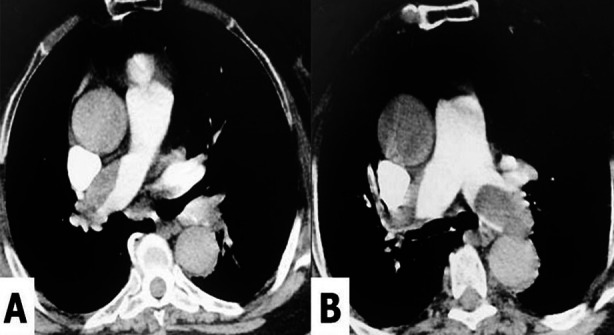
View of pulmonary embolism in computerized tomographic angiography.

In this study, patients diagnosed with PE in our hospital between March 1^st^, 2019 and February 28^th^, 2022 were retrospectively analyzed. The present study protocol was reviewed and approved by the Institutional Review Board (approval No. 2020/37). Patients, who were diagnosed with PE for the first time and over 18 years of age, were included in the study. Patients with the diagnosis of PE and under the age of 18 years old were excluded. The data of the patients was obtained from the patient files. Patients, diagnosed with PE, via computerized tomography were included in the study (Fig.1). Hospitalization rates, referral rates, treatment approaches and early-term outcomes were evaluated.

Our hospital is a training and research hospital that was established on February 1^st^, 2018 and is located in a region serving 2 million inhabitants. To act faster evaluation and treatment of PE, a PERT was established on November 15^th^, 2019, led by the cardiovascular surgery service, including physicians from the emergency medicine, chest diseases, anesthesia and reanimation, cardiology and radiology clinics. After the PERT was established, a “PE awareness” meeting was held to increase awareness of this team in our hospital. In this meeting, the importance of early diagnosis of PE, its clinical presentation, patient’s evaluation, treatment methods and long-term complications of untreated patients were focused. In this way, the hospital staff were informed about the presence of the PERT, which provides rapid consultation at any time of the day in case of suspected PE.

Patients, suspected or diagnosed with PE were consulted with PERT. Diagnosis of PE was confirmed by computerized tomographic angiography (CTA). In these patients echocardiography was performed. Pulmonary embolism was classified according to clinical and laboratory findings by the PERT. Patients were enrolled in four risk groups regarding their clinical status. Patients with normal blood pressure without right ventricular dysfunction, low risk; those with evidence of right ventricular dysfunction or high troponin levels (≥14 pg/mL for patients aged <75 years and ≥45 pg/mL for those ≥75 years), intermediate-low risk; those with signs of right ventricular dysfunction (right ventricular dilatation with the ratio of right ventricular diameter / left ventricular diameter ≥ 0.9 or ≤ 45% right ventricular ejection fraction) and high troponin levels, intermediate-high; patients with hemodynamic instability, high risk.^7^,^8^

Anticoagulant therapy was preferred for intermediate-low and low-risk patients. Catheter-directed thrombolysis was performed in the patients with intermediate-high and high risk. Patients, who decided to be hospitalized were followed up in the cardiovascular surgery clinic in the absence of active infection. In case of an infective process accompanying PE, patients were hospitalized in the intensive care unit or internal medicine clinics.

EkoSonic endovascular system (EKOS) (Boston Scientific, USA), Viper thrombolytic catheter (Invamed, Turkey), and angiojet thrombectomy system (Boston Scientific, USA), were applied for CDT. During the EKOS treatment or thrombolytic catheter, in unilateral embolism, following pulmonary artery catheterization, 6-mg bolus tPA was followed by one mg/h infusion. A loading dose of 3-mg from both pulmonary arteries was followed by 0.5 mg/h infusion in bilateral embolism. The treatment was discontinued at the 24^th^ hour. Catheters were removed five minutes after the termination of the CDT and routine anticoagulant treatment was initiated one hour after the removal of the catheter. Control echocardiography was done 48 hours after the treatment to evaluate right ventricular function.

Statistical analysis was performed with the IBM Statistical Package for the Social Sciences 24.0 (SPSS 24.0, SPSS Inc., Chicago, IL). N (%) was used for categorical variables and mean ± SD for continuous variables.

## RESULTS

There were ninety-eight PE patients aged older than 18 years who consulted PERT during the study period. The mean age of the patients was 62.8±16.4 and 59% were male. Nine patients had a fracture, twelve patients had recently Covid-19 infection and six patients had a history of long-term traveling. Twenty-nine patients had a proved deep venous thrombosis (Table-I).

**Table-I:** Demographics and clinical approach of the patients.

	Patients (n=98)
Age (years)	62.8±16.4
***Gender***	
Male	58 (59.3%)
Female	40 (40.8%)
***Risk Factors***	
Deep Venous Thrombosis	29 (29.6%)
Fracture	9 (9.2%)
History of travel	6 (6.1%)
Immobilization	4 (4.1%)
History of Surgery	5 (5.1%)
History of Covid-19 infection	12 (12.2%)
History of Cancer	9 (9.2%)
***Degree of Pulmonary embolism***	
Intermediate low	52 (53.1%)
Intermediate high	33 (33.7%)
High	13 (13.3%)
Refer to another hospital	10 (10.2%)
Hospitalization	58 (59.2%)
Advanced treatment procedure	37 (37.8%)

All patients with intermediate-low risk were treated medically. Totally ten patients were referred to another hospital and eight of them were before PERT establishment. While six of seven patients with intermediate-high or high risk were transferred to another hospital before the PERT, all the risky patients were hospitalized after the PERT. The hospitalization rate of the patients was 59.2% and advanced treatment procedures were applied in 37.8%. Systemic thrombolytic therapy was performed on two patients. One patient with metastatic brain tumor was treated with low-molecular-weight-heparin. Catheter-directed procedures were performed in 37 patients. Pharmacomechanical thrombectomy was applied in a patient with massive PE and a history of cranial bleeding five days before the diagnosis of PE. Catheter directed thrombolysis was performed via EkoSonic endovascular system in 27 patients and infusion catheter in 9 patients (Table-II). After the establishment of PERT mean time from diagnosis to reperfusion was found as 243 minutes.

**Table-II:** Treatment procedures of hospitalized patients.

	Intermediate-low (n = 52)	Intermediate-high (n = 29)	High (n=11)
Medical treatment (n)	52	3	0
Anticoagulant treatment	52	1	-
Systemic thrombolytic	0	2	-
CDT (n)	0	26	11
EkoSonic endovascular system (n)	-	19	8
AngioJet thrombectomy (n)	-	0	1
Thrombolytic catheter (n)	-	7	2

In the follow-up period, there was one pericardial effusion that necessitated operation and one mortality. Patient with a history of prostate cancer died due to sudden respiratory arrest, 13 hours after the initiation of CDT. Relief of right ventricular function was determined by post-procedural echocardiography in all the patients that were treated with ST or CDT. The ratio of right ventricular diameter to left ventricular diameter was decreased from 1.09±0.12 to 0.85±0.07. There was no re-hospitalization and mortality in 30-day follow-up period.

## DISCUSSION

Pulmonary embolism may be treated medically or by interventional procedures. Conventional medical treatment is prone to be insufficient, especially in the treatment of intermediate-high and high PE. Systemic thrombolytic or CDT therapy, which is increasingly popular and reliable, might increase survival and quality of life in these patients. Treatment with CDT concludes 25% decrease in the RV/LV diameter, 30% decrease in pulmonary artery obstruction in the first 48 hours.^9^ Planning and performing these treatment methods by an experienced PERT contributes to the determination of patient-specific treatment by facilitating the access of the patient to treatment. PERTs established in hospitals might help fast and effective treatment. As it might be clearly understood by our study that, PERT has an important role in the differential diagnosis of patients with the symptoms of chest pain and dyspnea. Evaluation of patients with PE by PERT allows patients to use advanced treatment procedures and provides the opportunity for early reperfusion with advanced treatment methods. This prevents chronic thromboembolic pulmonary hypertension in the long term and increases the quality of life of patients.

Due to the heterogeneity of patients presenting with PE variety of treatment methods are available. But due to a lack of consensus in guidelines; PERTs has emerged to overcome this deficiency.^5^ Experts in the field of emergency medicine, cardiovascular surgery, cardiology, chest diseases, anesthesiology, radiology, hematology clinics can contribute to PERTs according to the patient’s structure and the experience of the relevant clinics.^10^ PERTs also provide effective triage for PE patients, stabilization and determination of the most effective treatment method i.e. medical therapy, catheter-directed treatment or surgery).^11^ In our hospital, PERT was established under the leadership of the cardiovascular surgery department, experienced in advanced treatment techniques for PE. During this period, we realized that patients were hospitalized without being referred to other centers and the rate of interventional treatment applications increased.

In the treatment of PE, anticoagulation continues to be the main treatment for most patients. But alternative treatment methods cannot be applied in primary care centers in the presence of massive embolism or contraindications to anticoagulation.^4^ Considering that approximately 50% of deaths due to PE occur within the first 72 hours, referral of patients to experienced centers where alternative treatment methods might be applied is of great importance.^1^ It has been reported that awareness of PE and risk determination in PE increases with education.^12^ Therefore, after the establishment of PERT in our hospital, we organized a symposium to introduce our team and update the knowledge of the PE of health professionals including recent guidelines and recent treatment methods. Triage, diagnosis and treatment of patients with PE became to be faster than before with increased awareness. In the earlier period, patients with pneumonia or other respiratory disease consulted to PERT, however, with the increasing experience over time, only PE patients began to be consulted.

PERT decreases major or clinically insignificant bleeding, increases the usage of ST / CDT and decreases 30-day mortality in patients with intermediate and high risk.^5^ After the establishment of PERT, increased usage of extra-corporal membrane oxygenation (1.7% vs 7.89%), increased usage of CDT (1.8% vs 46.3%); decreased intensive care unit (ICU) staying time (6.9 ± 9.4 vs 4.4 ± 5.1 days) and hospital staying time (9.2 ± 16.1 vs 6.3 ± 7.4 days) were reported.^13^ Kabrhel et al.^10^ declared that the number of patients consulted every 6 months after the PERT team was established increased by 6%. Pulmonary embolism was detected in 80% of the patients consulted, and approximately 70% of them had submassive and massive pulmonary embolisms; however, 10% of the patients had undergone ST or CDT.^10^ Sista et al.^14^, applied CDT to 27% of their patients and ST therapy to 7% of their patients whom they consulted with PERT. Carroll et al.^1^ performed CDT on 11% of 70 patients evaluated with PERT and ST therapy 18% of them. Since we included the patients with the diagnosis of PE in this retrospective study, we did not have the opportunity to examine patients who were consulted with PERT and no PE. Catheter-directed procedures were applied to more than 1/3 of our patients. We prefer firstly EkoSonic endovascular system as CDT. In the difficulty to reach this system, we use an infusion catheter.

Confirmed PE are mostly (60%) diagnosed at emergency service and massive PE is most common (65%) in the ICU.^15^ Most of the emergency (54.8%) and inpatient wards (55%) are submassive embolisms. Thrombolysis or thrombectomy usage was reported as 33.3% in ICU patients, 19.6% in emergency service patients, and 8.2% in inpatient wards.^15^ In a study examining patients diagnosed with PE in the emergency department; it has been reported that the use of advanced treatment methods in the treatment of submassive and massive pulmonary embolism increased from 15% to 32% after the PERT.^16^ Patients with submassive PE and findings of RV dysfunction have a high risk for deterioration and need more aggressive treatment.^17^ However, ST is restricted in this group due to hemorrhagic complications including intracranial hemorrhage.^3^,^18^ Surgical embolectomy is suggested in only massive PE or the failure of ST or CDT, since it has 13% mortality risk.^2^,^3^ Currently, CDT is seen as the first treatment option for the treatment of submassive high-risk PE.

It was reported that the time from triage to diagnosis was reduced by 45%, and the time from diagnosis to the initiation of treatment was reduced by 58% with PERT teams.^16^ In another study in which 321 patients were evaluated, it was reported that the ICU staying time was decreased from five days to two days, and the reperfusion rate increased from 30% to 92% with PERT.^4^ In our study, patients diagnosed with PE in the emergency department were examined. There was a significant increase in hospitalization rates (from 21% to 59.8%) and use of CDT (from 7% to 37.8%) after the PERT evaluation. The time from diagnosis to reperfusion (243 minutes) was similar to the recent articles.^4^

Right ventricular dysfunction is prognostic value in patients with PE. Hospital mortality rate was reported as 6.6% in the patients with a right ventricular to a left ventricular ratio higher than 0.9.^19^ In the follow-up period, the mortality rate in three months was found as 17%, if this ratio was more than 1.5.^20^ Right ventricular dysfunction recovers in 48 hours with thrombolytic therapy.^6^,^18^ In our patients we observed 22% decrease in the ratio of right ventricular diameter to the left ventricular diameter at 48^th^ hour. This decrease was associated with the regression of clinical symptoms.

### Limitations of the study:

The mortality rates and treatment methods of the patients referred to an external center and patients who were not consulted with PERT or patients who were consulted with PERT without PE could not be reached. Prospective studies including the changes in right ventricular overloading and comparison of different treatment approaches will emphasize the importance of PERT.

## CONCLUSION

Treatment of pulmonary embolism still varies according to clinical experience. In the 2019 ESC guideline, ST is recommended as Class-1 indication, CDT as Class-2a indication in the treatment of intermediate-high and high-risk PE.^7^ In patients with PE, catheter-directed low-dose tPA infusion might be preferred instead of high-dose ST due to accompanying risk factors. With the increased experience of CDT, more patients will have a chance to reach advanced treatment options. The establishment of PERT with experienced specialists helps faster triage of patients with PE and prevents right ventricular dysfunction with early reperfusion. Additionally, it decreases the rates of mortality and morbidity and improves clinical outcomes with increasing quality of life in the long term.

### Authors’ Contribution:

**YO:** Conceptualization, investigation, accountable for the accuracy or integrity of the work, preparing the manuscript.

**MU:** Data curation, methodology, accountable for the accuracy or integrity of the work, preparing the manuscript.

**CO:** Formal analysis, supervision, review.

**EY:** Methodology, validation, review.
